# Exploring self-directed learning practices through ChatGPT among non-native Arabic learners at a Saudi university

**DOI:** 10.3389/fpsyg.2026.1889915

**Published:** 2026-08-05

**Authors:** Yasir A. Alsamiri, Abdulmohsin A. Alshehri

**Affiliations:** 1 Department of Education, Islamic University of Madinah, Medina, Saudi Arabia; 2 Department of Languages and Translation, Faculty of Arts and Humanities, Taibah University, Medina, Saudi Arabia

**Keywords:** Arabic language learning, artificial intelligence in education, ChatGPT, learner autonomy, non-native Arabic learners, self-directed learning

## Abstract

**Introduction:**

This study examined how non-native learners of Arabic at a Saudi university used ChatGPT as part of their self-directed learning practices. It focused on the role of ChatGPT in supporting Arabic language development and fostering motivation for independent learning.

**Methods:**

Adopting a qualitative research design, the study collected data through semi-structured, in-depth interviews with six learners from diverse ethnic backgrounds. The data were analyzed using thematic analysis to identify recurring themes and patterns in participants’ experiences.

**Results:**

The findings revealed that ChatGPT played a meaningful role in supporting learners’ Arabic language acquisition. Participants reported improvements in vocabulary, grammatical knowledge, and reading comprehension, highlighting the tool’s value as a readily accessible learning resource. In addition, ChatGPT appeared to strengthen learners’ motivation for autonomous learning by encouraging greater independence, promoting active engagement with the language, and allowing learners to tailor their learning experiences to their individual needs.

**Discussion:**

Overall, the study demonstrates the potential of AI-powered tools such as ChatGPT to enhance Arabic language learning and support the development of learner autonomy.

## 1 Introduction

Arabic is one of the most widely spoken languages in the world, with more than 400 million speakers across the Middle East, North Africa, and numerous diaspora communities. In addition to its status as an official language of international organizations such as the United Nations and UNESCO, Arabic occupies a unique place in global culture because it is the language of the Holy Qur’an and a central vehicle of Islamic scholarship and civilization. Over the past decade, interest in learning Arabic as a foreign language (AFL) has grown considerably. Students pursue Arabic for a variety of reasons, including academic study, career opportunities, religious purposes, and intercultural communication (Alshehri et al., 2025; Ritonga et al., 2024; Soliman and Khalil, 2024).

Despite this growing interest, learning Arabic presents a number of challenges for non-native speakers. The language is characterized by a rich morphological system, complex grammatical structures, and a diglossic nature that requires learners to navigate both Modern Standard Arabic and spoken varieties. In addition, the Arabic script, right-to-left writing system, and extensive vocabulary often create difficulties for learners, particularly in reading and writing. As a result, many students require support that extends beyond classroom instruction in order to develop proficiency and maintain steady progress (AlAwfi, 2024; Almelhes, 2024).

In response to these challenges, language educators and researchers have increasingly turned their attention to technology-enhanced learning. Digital tools have long been used to supplement language instruction, but recent developments in artificial intelligence (AI) have introduced new possibilities for personalized and interactive learning experiences. Unlike traditional language-learning platforms that rely on fixed exercises and preprogrammed responses, AI-based systems can adapt more readily to individual learner needs and provide immediate feedback, creating opportunities for continuous practice and engagement (Qpilat and Salam, 2023; Li et al., 2022).

Among these developments, ChatGPT has emerged as one of the most widely discussed AI tools in education. Built on large language model technology, ChatGPT can generate human-like responses, engage users in extended conversations, and provide explanations on a wide range of topics. These capabilities have attracted considerable attention from educators, researchers, and students alike. In language-learning contexts, ChatGPT can serve multiple roles simultaneously: a tutor, conversation partner, writing assistant, and source of feedback. Learners can ask questions about grammar, explore vocabulary meanings, request examples of language use, and receive support with reading and writing tasks in real time (Kohnke et al., 2023; Jiang et al., 2022).

For learners of foreign languages, one of the most appealing features of ChatGPT is its accessibility. Students can interact with the system whenever they need assistance, regardless of time or location. This flexibility allows learners to practice language skills outside the classroom and at their own pace. Such opportunities are particularly valuable for individuals who may have limited access to native speakers or personalized instruction. Consequently, researchers have begun examining how ChatGPT influences language-learning outcomes, learner engagement, and independent learning behaviors across different educational settings (Boudouaia et al., 2024; ZainEldin et al., 2024).

## 2 Theoretical framework

This study is guided by the concepts of self-directed learning and learner autonomy, two perspectives that emphasize the active role learners play in managing their own educational development.

Knowles (1975) describes self-directed learning as a process in which learners assume responsibility for identifying their learning needs, setting goals, locating resources, choosing learning strategies, and evaluating outcomes. Rather than depending entirely on teacher guidance, learners become active participants in shaping their learning experiences. The concept has become increasingly relevant in contemporary educational contexts, where digital technologies provide unprecedented access to information and learning resources.

Closely related to self-directed learning is learner autonomy. Holec (1981) defines learner autonomy as the ability to take responsibility for one’s own learning decisions. Autonomous learners determine what they want to learn, how they intend to learn it, and how they will assess their progress. Benson (2013) argues that autonomy is particularly important in second- and foreign-language learning because meaningful language development often requires practice, exposure, and interaction beyond formal classroom instruction.

These perspectives provide a useful foundation for understanding the potential role of ChatGPT in language learning. Through immediate access to explanations, personalized feedback, and opportunities for ongoing practice, ChatGPT may help learners take greater control over their language-learning journeys. Rather than waiting for teacher assistance, learners can seek clarification independently, experiment with language use, and monitor their own progress. In this sense, ChatGPT can be viewed not simply as a technological tool but as a potential facilitator of self-directed and autonomous learning.

### 2.1 ChatGPT and language learning

The increasing interest in artificial intelligence has led researchers to reconsider how technology can support language learning both inside and outside the classroom. Among the various AI-driven innovations, conversational systems such as ChatGPT have attracted particular attention because they offer learners opportunities for interaction that extend beyond the static exercises typically found in conventional language-learning applications. Rather than simply delivering pre-programmed responses, ChatGPT can engage users in extended dialogue, respond to individual questions, and generate personalized feedback. These capabilities have prompted many educators and researchers to view the technology as a potentially valuable resource for language learning. At the same time, growing enthusiasm for AI has sometimes outpaced the empirical evidence, making it important to examine not only what ChatGPT can do, but also how learners actually use it and what educational value those interactions provide.

Much of the existing research has highlighted the versatility of ChatGPT in supporting different aspects of language development. Learners commonly use the tool to obtain grammatical explanations, clarify unfamiliar vocabulary, practice conversational exchanges, and receive assistance with reading and writing tasks (Kohnke et al., 2023; Jiang et al., 2022). Its appeal lies largely in the immediacy of its responses and the flexibility of the learning experience it offers. Rather than waiting for instructor feedback or searching through multiple learning resources, learners can interact with ChatGPT whenever questions arise. This accessibility has led some scholars to suggest that AI-powered systems may help bridge gaps between classroom instruction and independent study.

Writing has emerged as one of the most frequently studied areas of ChatGPT use. Foreign language learners often struggle with generating ideas, organizing content, and maintaining grammatical accuracy, particularly when writing independently. Research suggests that ChatGPT can support learners during various stages of the writing process by offering suggestions, identifying language errors, and providing feedback on clarity and organization (Mohideen, 2024; Boudouaia et al., 2024). However, the potential value of such support may lie less in the corrections themselves and more in how learners engage with them. Simply accepting AI-generated revisions may do little to promote long-term language development. The educational benefits are more likely to emerge when learners critically evaluate the feedback, compare alternatives, and reflect on the linguistic choices being suggested. In this sense, ChatGPT may function most effectively as a scaffold for learning rather than as an automated solution to writing difficulties.

A similar pattern can be observed in vocabulary learning. Researchers have reported that learners use ChatGPT to obtain definitions, explore word usage, and generate contextualized examples. Such interactions can expose learners to language in ways that traditional vocabulary lists often cannot. Nevertheless, vocabulary acquisition is a gradual process that requires repeated exposure and meaningful use across different contexts. While ChatGPT can facilitate this process by generating varied examples and explanations, exposure alone does not guarantee learning. As several scholars have noted, the effectiveness of AI-mediated vocabulary practice depends largely on the extent to which learners actively engage with new lexical items rather than merely consuming information provided by the system.

Beyond writing and vocabulary development, ChatGPT has been used to support reading comprehension and conversational practice. Learners frequently ask the system to summarize texts, explain difficult passages, or engage in simulated dialogues in the target language. These activities can create valuable practice opportunities, particularly for learners who have limited access to native speakers or individualized instruction. Yet it is important to recognize that interactions with AI differ fundamentally from authentic human communication. Although ChatGPT can simulate conversation remarkably well, it does not participate in communication as a social actor with genuine intentions, emotions, or cultural experiences. Consequently, while AI-based interactions may complement language practice, they cannot fully replicate the social and cultural dimensions of communication that are central to language use.

The literature has also drawn attention to the motivational benefits associated with ChatGPT. Many learners describe the tool as approachable, readily available, and non-judgmental, characteristics that may lower anxiety and encourage experimentation with the target language (ZainEldin et al., 2024; El Zahraa, 2025). This is particularly relevant in foreign-language contexts where learners may hesitate to participate due to concerns about making mistakes. However, motivation should not be viewed as an automatic outcome of technology use. Initial enthusiasm for new technologies can diminish over time, and learners may ultimately judge the value of a tool according to how well it supports their learning goals. Therefore, sustained engagement is likely to depend not only on the novelty of ChatGPT but also on learners’ perceptions of its usefulness and relevance to their language-learning needs.

From a theoretical perspective, ChatGPT appears to align closely with principles of self-directed learning and learner autonomy. Its availability enables learners to seek information independently, address gaps in their knowledge, and engage in self-paced practice. These features have led some researchers to argue that AI technologies can empower learners to take greater responsibility for their own learning. Yet autonomy involves more than having access to resources. As Benson (2013) argues, autonomous learning requires learners to make informed decisions, evaluate information critically, and regulate their learning processes. Access to ChatGPT may create opportunities for autonomous learning, but whether those opportunities are realized depends largely on learners’ ability to use the technology strategically and critically.

At the same time, the limitations of ChatGPT have generated substantial discussion within the literature. One concern involves the possibility of inaccurate or fabricated information, often referred to as AI hallucinations. Although the system typically produces fluent and convincing responses, fluency should not be equated with accuracy. Learners who lack sufficient linguistic or subject knowledge may struggle to identify errors, potentially resulting in misunderstandings or the reinforcement of incorrect information (Kasneci et al., 2023). This challenge highlights the importance of developing critical digital literacy skills alongside the use of AI technologies.

Another concern relates to learner dependence on AI-generated support. While immediate assistance can facilitate learning, excessive reliance on automated feedback may reduce learners’ engagement with the cognitive processes that underpin language development. Language learning requires sustained effort, problem-solving, and reflection. If learners routinely delegate these processes to AI systems, they may become less active participants in their own learning. Consequently, the educational value of ChatGPT should not be measured solely by the quality of the responses it provides, but also by the extent to which it encourages meaningful learner engagement.

Overall, the existing literature presents a more nuanced view of ChatGPT than either enthusiastic advocacy or outright skepticism suggests. The technology offers considerable potential to support language learning by expanding access to practice, feedback, and learning resources. However, its effectiveness cannot be understood independently of the learners who use it and the educational contexts in which it is employed. Rather than viewing ChatGPT as a substitute for teachers or established pedagogical practices, it may be more productive to regard it as a resource whose value depends on thoughtful, critical, and purposeful use. Such a perspective is particularly relevant for learners of Arabic as a foreign language, where questions concerning independent learning, language practice, and learner autonomy remain underexplored.

### 2.2 Research gap

The growing interest in artificial intelligence has generated an expanding body of research on its applications in language education. However, despite the increasing number of studies examining AI-assisted language learning, several important questions remain insufficiently explored. One notable issue concerns the linguistic contexts represented in the existing literature. Much of the current research has focused on English and other widely taught foreign languages, while Arabic has received comparatively limited attention. This imbalance is significant because Arabic presents learners with a set of linguistic challenges that differ in important ways from those associated with many other languages. Its complex morphology, diglossic nature, and distinctive writing system shape the learning experience in ways that may influence how learners interact with AI-based tools. As a result, findings from studies conducted in other language contexts cannot simply be assumed to apply to learners of Arabic.

Another limitation concerns the settings in which AI-supported learning has been investigated. Existing studies have largely examined learner achievement, learner attitudes, or the integration of AI technologies within formal instructional environments. While these areas have contributed valuable insights, they provide only a partial picture of how learners engage with AI in their everyday learning practices. Language development often extends beyond the classroom, particularly in foreign-language contexts where learners must create opportunities for additional exposure and practice. Yet relatively little is known about how learners use tools such as ChatGPT independently, what purposes these tools serve in their learning routines, and how they contribute to ongoing language development outside teacher-directed environments.

The experiences of learners studying Arabic in Saudi Arabia represent another area that remains underexplored. Saudi universities have long played a central role in providing Arabic-language education to international students and have increasingly embraced digital and emerging educational technologies. Despite this context, relatively few studies have examined how non-native learners of Arabic in Saudi higher education engage with AI-powered learning tools. Consequently, there is limited empirical evidence regarding how these learners incorporate ChatGPT into their learning practices, what types of language-learning activities they undertake through the platform, and how they evaluate its contribution to their linguistic development.

A further issue is that much of the literature discusses artificial intelligence in broad terms, often treating AI as a single educational phenomenon. Such an approach can obscure important differences between specific technologies and how they are used by learners. ChatGPT, for example, differs from many previous language-learning tools because of its conversational capabilities, accessibility, and capacity to generate individualized responses. These characteristics have the potential to shape learning experiences in ways that may not be captured in studies examining AI as a general category. A more focused investigation of ChatGPT is therefore needed to better understand its particular role in supporting language learning, learner autonomy, and motivation among learners of Arabic as a foreign language.

Taken together, the existing literature suggests that the educational potential of ChatGPT in Arabic language learning remains only partially understood. While research has documented the growing use of AI in language education, less attention has been paid to how learners employ such technologies as part of their self-directed learning practices, particularly in the context of Arabic as a foreign language. Furthermore, little is known about how learners evaluate the role of ChatGPT in supporting their language development, motivation, and autonomy. By addressing these issues, the present study seeks to contribute to a more nuanced understanding of how generative AI is shaping independent language learning and to provide insights that may inform the effective integration of AI-supported learning within Arabic language education.

### 2.3 The current study

Building on the gaps identified in the literature, the present study examines how non-native learners of Arabic use ChatGPT as part of their language-learning practices beyond formal classroom instruction. Although recent research has documented the growing role of artificial intelligence in language education, relatively little is known about how learners of Arabic employ ChatGPT to support self-directed learning or how they perceive its contribution to their language development. Guided by the theoretical perspectives of self-directed learning and learner autonomy, this study seeks to explore learners’ experiences with ChatGPT and to better understand the role the technology plays in supporting independent language learning. Rather than focusing solely on learning outcomes, it considers how learners engage with the technology, the purposes for which they use it, and the role it plays in supporting their learning goals. Particular attention is given to how ChatGPT is used to develop different aspects of Arabic proficiency, including reading comprehension, writing, vocabulary knowledge, and grammatical competence.

The current study also investigates learners’ perceptions of ChatGPT as a language-learning resource. While recent discussions of generative AI have highlighted its potential educational benefits, learners’ experiences are likely to be more complex and nuanced. For this reason, this study explores not only the perceived advantages of using ChatGPT but also the challenges and limitations learners encounter. In doing so, it seeks to develop a more balanced understanding of the role that AI-powered technologies can play in language learning.

More specifically, the study addresses the following research questions:

1. What self-directed learning practices do non-native Arabic learners use with ChatGPT?
2. How do non-native Arabic learners perceive the impact of ChatGPT on their Arabic language development?
3. How does ChatGPT influence non-native Arabic learners’ motivation, autonomy, and literacy skills?

By focusing on learners of Arabic as a foreign language within a Saudi university context, the study contributes to an area that remains comparatively underexplored in the literature on AI-assisted language learning. Examining learners’ experiences can provide valuable insights into how generative AI technologies are being incorporated into independent language-learning practices and how learners make use of these tools to support their development. More broadly, the findings may contribute to ongoing discussions about the relationship between AI, learner autonomy, and self-directed language learning. They may also offer practical insights for educators, curriculum designers, and policymakers seeking to integrate AI technologies into Arabic language education in ways that support learner independence while maintaining the central importance of effective pedagogy and meaningful human interaction.

## 3 Methodology

### 3.1 Research design

This study employed a qualitative research approach using an exploratory case study design to examine how non-native learners of Arabic use ChatGPT as a self-directed learning tool at the first author’s university in Saudi Arabia. A case study approach was considered appropriate because it enables researchers to investigate a contemporary phenomenon within its real-life context and to gain a detailed understanding of participants’ experiences and perspectives (Yin, 2018).

The use of generative AI tools such as ChatGPT in Arabic language learning is still relatively new, and little is known about how learners use these tools independently outside formal classroom settings. For this reason, an exploratory design was selected. Rather than testing predetermined hypotheses, the study sought to understand participants’ experiences, perceptions, and learning practices and to identify aspects of ChatGPT use that may not yet be well documented in the literature.

The study was guided by an interpretive perspective, with the assumption that learners construct their own understanding of educational technologies through personal experience and interaction. Accordingly, the focus was on how participants made sense of ChatGPT and how they integrated it into their Arabic language learning practices.

The interview data were analyzed inductively, allowing themes to emerge from participants’ accounts rather than being organized according to predefined categories. Although the research questions provided a general direction for the analysis, the researchers remained open to identifying unexpected patterns and insights that arose from the data.

As with most qualitative case studies, the purpose of this research was not to produce statistically generalizable findings. Instead, the study aimed to develop a rich understanding of learners’ experiences within a particular educational context. By providing detailed descriptions of the participants and research setting, readers can determine the extent to which the findings may be relevant to similar contexts.

### 3.2 Participants

The study involved six non-native learners of Arabic from diverse linguistic and cultural backgrounds, including the Philippines, Indonesia, India, Malaysia, Uganda, and Pakistan. Participants were enrolled in different academic programs at the university, including Sharia, Theology and Da’wah, Arabic Language and Humanities Studies, and Law and Economics.

Participants were selected through purposive sampling because the study required individuals who had direct experience using ChatGPT for Arabic language learning. To be included in the study, participants had to meet three criteria. First, they had to be regular users of ChatGPT for at least 1 month prior to the study. Second, they needed to have used ChatGPT specifically to support their Arabic language learning. Third, they had to have completed at least 4 years of study at the university. These criteria were established to ensure that participants had sufficient experience with both Arabic language learning and the use of ChatGPT to provide informed and reflective accounts.

The sample consisted of four undergraduate students and two postgraduate students aged between 24 and 29 years (*M* = 26.3). Additional information, including Arabic proficiency level, length of Arabic study, and frequency of ChatGPT use, was also collected to provide a fuller understanding of participants’ backgrounds (see Table 1).

**Table 1:** Demographic profile of participants.

Participant	Nationality	College	University Level	Age	Arabic Proficiency	Years Studying Arabic	Frequency of ChatGPT Use
P1	Philippine	Sharia	Bachelor	24	Elementary	3 years	Daily
P2	Indonesia	Theology and Da’wah	Bachelor	26	Upper Intermediate	5 years	Daily
P3	India	Arabic Language and Humanities Studies	Bachelor	25	Proficient	8 years	A few times a week
P4	Malaysia	Law and Economics	Master	28	Advanced	6 years	Several times a week
P5	Uganda	Theology and Da’wah	Bachelor	26	Intermediate	4 years	Daily
P6	Pakistan	Law and Economics	Master	29	Advanced	7 years	Several times a week

The sample size was relatively small; however, this was consistent with the exploratory nature of the study and with the goals of qualitative case study research, where depth of understanding is prioritized over broad representation. Data collection continued until thematic saturation was reached. By the sixth interview, participants were describing similar experiences and no substantially new themes were emerging. At that point, the researchers concluded that sufficient data had been collected to address the research questions.

Although the findings offer valuable insights into learners’ experiences, they should be interpreted within the context of a small group of participants from a single institution. The intention is therefore not to claim broad generalizability but to provide a detailed understanding of a particular group of Arabic learners and their engagement with ChatGPT.

### 3.3 Instrument and data collection

Data were collected through semi-structured interviews. This method was chosen because it provides a balance between structure and flexibility, allowing participants to share their experiences in detail while giving the interviewer opportunities to explore issues that emerged during the conversation.

The interview guide was developed after reviewing literature related to self-directed learning, language learning technologies, artificial intelligence in education, and second-language acquisition. The questions were designed to encourage participants to reflect on how they used ChatGPT, the kinds of learning activities they engaged in, the benefits they perceived, and any challenges they encountered.

Before data collection began, the interview protocol was reviewed by a scholar in applied linguistics to assess the clarity and relevance of the questions. Minor revisions were made based on the feedback received. A pilot interview was then conducted with a student who met the study criteria but did not participate in the final sample. This helped ensure that the questions were understandable and capable of eliciting detailed responses.

The interviews were conducted face-to-face between March and April 2025. All interviews were carried out in Arabic to allow participants to express themselves comfortably and naturally. Each interview lasted approximately 30 min and was audio-recorded with participants’ permission.

Following the interviews, the recordings were transcribed verbatim and translated into English. Translation was undertaken by a proficient bilingual translator. To ensure accuracy, the translated transcripts were compared with the original Arabic recordings, and selected excerpts were back-translated to check for consistency in meaning. Any discrepancies were discussed and resolved during the review process.

To maintain consistency across interviews, all interviews were conducted by the second author. Throughout the data collection process, the researchers kept field notes and reflective memos to document observations, emerging ideas, and potential sources of researcher influence on the research process.

### 3.4 Data analysis

The interview data were analyzed using inductive thematic analysis (Braun and Clarke, 2022; Lochmiller, 2021). This approach was selected because it allows themes to emerge from participants’ experiences rather than imposing predetermined categories onto the data.

Analysis began with repeated readings of the transcripts to develop familiarity with the data. During this stage, the researchers noted initial impressions, recurring ideas, and potentially meaningful patterns. Listening to interview recordings alongside the transcripts helped ensure that important contextual details were not overlooked.

The next stage involved open coding. Segments of text that appeared relevant to the research questions were identified and assigned descriptive codes. Coding was conducted inductively, meaning that codes were derived from the data itself rather than from an existing theoretical framework.

Once the initial coding was complete, related codes were grouped together to form broader categories. The researchers then reviewed these categories across all interviews to identify patterns that captured shared experiences among participants. As the analysis progressed, some categories were combined, revised, or renamed to improve clarity and coherence.

The emerging themes were continuously compared against the original data to ensure that they accurately reflected participants’ accounts. Particular attention was given to similarities and differences across participants, allowing both common patterns and individual perspectives to be identified.

The analysis was conducted manually due to the relatively small size of the dataset. Working directly with the transcripts enabled the researchers to remain closely engaged with the data throughout the analytical process.

To enhance the credibility of the findings, both researchers independently reviewed the coding and thematic structure. The researchers then met regularly to discuss their interpretations and resolve any differences in coding. Through these discussions, a shared understanding of the data and final themes was reached.

### 3.5 Ethical considerations

Ethical approval for this study was granted by the Standing Committee for Research Ethics at a university affiliated with one of the researchers (Reference No. H-1445-5-26) before the commencement of data collection. All participants received information about the purpose of the study, the interview procedures, and their rights as research participants. Participation was entirely voluntary, and written informed consent was obtained from all participants prior to their involvement in the study. Participants were also informed that they could withdraw from the study at any stage without penalty.

To ensure confidentiality, pseudonyms were assigned to all participants, and any identifying information was removed from the interview transcripts and related records. Audio recordings, transcripts, and field notes were stored on an encrypted, password-protected device accessible only to the researchers. All data were treated as confidential and used solely for the purposes of this study. No information that could identify individual participants is reported in the findings.

### 3.6 Trustworthiness

Several strategies were employed to enhance the trustworthiness of the study.

To strengthen credibility, the researchers conducted in-depth interviews and used follow-up questions to encourage participants to elaborate on their experiences. Throughout the analysis, the interview recordings and transcripts were revisited repeatedly to ensure that interpretations remained closely connected to participants’ accounts. Participants were also invited to review summaries of their interviews and the researchers’ preliminary interpretations. Their feedback confirmed that the summaries accurately reflected their experiences and perspectives.

The researchers also engaged in ongoing discussions about coding decisions and theme development during the analytical process. In addition, an experienced qualitative researcher who was not directly involved in the study reviewed the developing analysis and provided feedback on the interpretation of the data. These discussions helped challenge assumptions and strengthen the overall rigor of the analysis.

To support dependability, the researchers maintained a detailed record of important decisions made during data collection and analysis. This documentation included interview notes, coding records, analytical memos, and reflections on the research process.

Confirmability was enhanced through reflexive practice. The researchers kept reflective notes throughout the study and regularly considered how their own experiences and assumptions might influence the interpretation of participants’ responses. Independent review of the coding and subsequent discussion also helped ensure that the findings remained grounded in the data.

Transferability was addressed by providing detailed descriptions of the participants, the research setting, and the procedures followed throughout the study. These descriptions enable readers to evaluate the relevance of the findings to other educational contexts.

## 4 Findings

This study explored how non-native learners of Arabic at a Saudi university use ChatGPT to support their self-directed learning. Analysis of the interview data revealed two overarching themes. The first theme relates to the use of ChatGPT for improving Arabic language skills, while the second concerns its role in enhancing motivation for self-directed learning. Within these two themes, six sub-themes emerged from participants’ experiences. Table 2 presents an overview of the themes and sub-themes identified through the analysis.

**Table 2:** Themes, sub-themes, and representative participant contributions emerging from the thematic analysis.

Theme	Sub-theme	Representative participants
Theme 1: Using ChatGPT to Improve Arabic Language Learning Skills	Vocabulary improvement	P2, P3, P4, P6
	Improving grammatical knowledge	P1, P4, P5, P6
	Improving reading comprehension	P2, P4, P5, P6
Theme 2: Using ChatGPT to Boost Students’ Motivation for Self-Directed Learning	Personal interaction and instant responses	P3, P5
	Immediate feedback	P1, P2, P4, P6
	Encouraging independence and self-directed learning	P3, P4

Overall, participants viewed ChatGPT as a valuable resource that supported both language development and independent learning. They described using the tool to improve their vocabulary, grammar, and reading comprehension, while also relying on it for immediate assistance when encountering learning difficulties. Beyond its linguistic benefits, participants highlighted the motivating effect of instant responses, corrective feedback, and continuous access to support. These features appeared to encourage learners to take greater responsibility for their own learning and engage more actively with Arabic beyond the classroom. These themes and sub-themes are discussed in detail below with supporting quotations from participants.

### 4.1 Theme 1: using ChatGPT to improve Arabic language learning skills

One of the most prominent themes emerging from the interviews was the contribution of ChatGPT to the development of Arabic language skills. All participants described incorporating the tool into their learning routines in different ways. Whether they were learning new vocabulary, clarifying grammatical rules, or trying to understand challenging texts, ChatGPT was frequently used as a source of explanation and guidance. Participants often referred to the convenience of accessing support whenever they needed it, allowing them to continue learning outside formal classroom settings.

#### 4.1.1 Sub-theme 1: vocabulary improvement

Vocabulary development was among the most commonly cited benefits of using ChatGPT. Participants explained that they frequently consulted the tool when they encountered unfamiliar words, wanted to understand different meanings, or needed examples of how words were used in context. Many felt that interacting with ChatGPT exposed them to a wider range of vocabulary than they would normally encounter during regular classroom activities. For example, P2 described how conversational exchanges with ChatGPT encouraged vocabulary growth:

By using ChatGPT to create vocabulary exercises through daily conversations with ChatGPT, students can naturally expand their vocabulary through the repeated use of words in different contexts (P2).

Similarly, P6 explained that seeing words used in multiple contexts made them easier to remember:

I like to ask ChatGPT about words that I do not understand well. I can get the meanings of the words, along with how they are used in different sentences, and this helps me remember them better (P6).

Several participants suggested that vocabulary learning often occurred naturally during their interactions with the tool. As P4 noted:

Whenever I interact with ChatGPT, I come across new words and expressions that I have not learned before, and this helps me increase my vocabulary (P4).

Vocabulary learning was also closely connected to broader language development. P3 highlighted this relationship when explaining:

Through interacting with ChatGPT, I learned more about grammar rules and sentence structure in Arabic. It’s not just about learning vocabulary, but also about learning how to organize words correctly in sentences (P3).

Overall, participants viewed ChatGPT as a useful resource for expanding their vocabulary through exposure to meanings, examples, and contextualized language use.

#### 4.1.2 Sub-theme 2: improving grammatical knowledge

Grammar learning emerged as another important area in which participants found ChatGPT helpful. Many participants explained that they used the tool whenever they encountered difficulties with Arabic grammatical rules, particularly concepts that they considered complex or confusing. Rather than simply providing answers, ChatGPT was valued for its ability to explain rules and demonstrate how they function in authentic language use. For instance, P1 emphasized the usefulness of examples in understanding grammatical concepts:

Students can request examples of how different grammatical rules are used in sentences, which helps in understanding the practical application of the rules (P1).

Likewise, P4 described how the tool supported the learning of more challenging grammatical structures:

In fact, I use ChatGPT especially when I want to learn how to conjugate irregular verbs in correct sentences (P4).

Participants also appreciated the role of ChatGPT in identifying and correcting mistakes. For P5, grammar learning often occurred through receiving explanations about language errors:

When I make mistakes in writing or speaking, ChatGPT helps me identify and correct those mistakes, which improves my understanding of grammar (P5).

Similarly, P6 described how detailed explanations contributed to a clearer understanding of sentence construction:

I can ask ChatGPT to explain the grammatical structure of a sentence step by step, and this makes it easier to understand how the sentence is formed (P6).

These accounts suggest that participants viewed ChatGPT as more than a reference tool. It was frequently used as a learning companion that helped them explore grammatical concepts, verify their understanding, and learn from their mistakes. Such practices reflect learners’ efforts to take an active role in addressing their own learning needs.

#### 4.1.3 Sub-theme 3: improving Reading comprehension

Reading comprehension was another area in which participants perceived considerable benefits from using ChatGPT. Learners described turning to the tool when they encountered difficult words, complex sentence structures, or passages that required deeper interpretation. For many, the ability to ask questions about a text and receive immediate clarification made reading Arabic materials more manageable and enjoyable. For example, P2 explained how ChatGPT could assist learners in understanding texts:

Students can submit texts in Arabic (such as excerpts from books or articles) to ChatGPT and request an explanation of difficult words or an interpretation of complex sentences (P2).

Participants also highlighted the role of ChatGPT in helping them understand literary and cultural meanings. P4 explained:

I can interact with literary texts, so non-native Arabic speakers can study Arabic literature or short stories and ask ChatGPT to explain the cultural or literary messages behind the texts (P4).

P5 provided a similar example:

If a non-native Arabic speaker presents a poem like verses from the poem ‘If the people one day desire life’, they can ask ChatGPT about the meaning of difficult words or inquire about the literary and cultural message behind these verses (P5).

For P6, contextual explanations played a particularly important role in comprehension:

When reading novels or articles, I can ask ChatGPT about the meaning of a sentence in its context, which helps me understand the text better (P6).

Participants’ accounts suggest that ChatGPT supported reading comprehension on multiple levels. In addition to helping learners understand vocabulary and sentence structures, it also enabled them to engage with broader cultural and literary dimensions of Arabic texts. This allowed learners to move beyond surface-level understanding and develop deeper interpretations of what they were reading.

### 4.2 Theme 2: using ChatGPT to boost students’ motivation for self-directed learning

Alongside its contribution to language development, ChatGPT was also viewed as an important source of motivation for self-directed learning. Participants consistently described the tool as something that encouraged them to study independently and engage more actively with Arabic outside formal learning environments. The accessibility of ChatGPT, together with its ability to provide immediate support, appeared to make learners feel more confident in taking control of their learning.

Three interconnected factors emerged from the interviews: the personal and interactive nature of communication with ChatGPT, the provision of immediate feedback, and the encouragement of greater independence in learning.

#### 4.2.1 Sub-theme 1: personal interaction and instant responses

A recurring idea across participant responses was the value of receiving immediate answers to questions. Participants explained that instant access to explanations helped them overcome learning obstacles without interrupting their study sessions. Rather than postponing questions until they could consult a teacher, learners were able to obtain clarification immediately and continue learning. P3 illustrated this experience with the example of learning grammar:

If a non-native Arabic speaker studies Arabic grammar and encounters difficulty understanding the rule of tanween (nunation), they can ask an immediate question to ChatGPT about how to use tanween in different sentences. By receiving an instant answer, the student feels confident and accomplished, which boosts their motivation to continue learning the language rules (P3).

Similarly, P5 described how immediate responses encouraged continued engagement with learning:

Whenever I have a question about Arabic language or culture, I can ask ChatGPT and receive an answer immediately. This makes learning easier and encourages me to keep studying (P5).

Beyond the speed of responses, participants frequently referred to the interactive nature of their conversations with ChatGPT. P3 explained:

ChatGPT provides non-native Arabic speakers with a sense of continuous interaction, which enhances their feeling of engagement and progress in learning the language (P3).

A similar sentiment was expressed by P2:

The interaction feels similar to having someone available all the time to answer questions and explain language concepts whenever needed (P2).

For many learners, this constant availability reduced frustration and created a sense of support throughout the learning process. The combination of rapid responses and interactive communication appeared to play an important role in sustaining motivation and encouraging continued engagement with Arabic learning activities.

#### 4.2.2 Sub-theme 2: immediate feedback

Another important motivational factor identified by participants was the availability of immediate feedback. Learners frequently described how ChatGPT could quickly identify errors and provide corrections, helping them recognize weaknesses and make improvements promptly. For instance, P1 explained:

When students write texts or sentences in Arabic, they can receive immediate corrections from ChatGPT. These corrections give students a sense of continuous progress and improvement, motivating them to continue learning (P1).

Similarly, P4 highlighted the encouraging nature of corrective feedback:

When ChatGPT shows me my mistakes and explains how to correct them, I feel that I am improving, and this encourages me to continue practising Arabic (P4).

For P2, feedback was closely connected to confidence building:

The feedback helps students understand what they did wrong and how to improve. This makes them feel more confident in using Arabic (P2).

Likewise, P6 appreciated that the corrections were accompanied by explanations:

ChatGPT not only corrects errors but also explains the reasons behind the corrections, which helps me avoid making the same mistake again (P6).

Several participants also noted that receiving feedback from ChatGPT felt less intimidating than receiving feedback in other situations. The absence of judgment appeared to make learners more willing to experiment with the language and learn from their mistakes. As a result, feedback was perceived not only as a means of improving language performance but also as an important source of encouragement and confidence.

#### 4.2.3 Sub-theme 3: encouraging Independence and self-directed learning

The final sub-theme relates to learner autonomy. Participants consistently described ChatGPT as a tool that enabled them to become more independent in their studies. By providing explanations, examples, and guidance whenever needed, the tool reduced learners’ dependence on teachers and allowed them to address difficulties on their own. As explained by P3:

ChatGPT enhances students’ independence and encourages them to learn without fully relying on traditional teachers. Students feel that they are capable of learning Arabic on their own, which boosts their confidence (P3).

Similarly, P4 reflected on how the tool encouraged exploration and initiative:

From my experiences with using ChatGPT, by interacting with the tool, I can discover new vocabulary and topics on my own, which enhances my motivation for exploration and self-directed learning (P4).

The flexibility offered by ChatGPT was also highlighted by P2:

I can study whenever I want and focus on the language areas that I personally need to improve. This makes me more responsible for my own learning (P2).

Likewise, P6 described using the tool as a means of solving problems independently:

When I face difficulties in grammar or vocabulary, I can use ChatGPT to find explanations by myself instead of waiting for help from others (P6).

Participants further explained that this sense of independence encouraged them to engage with Arabic more frequently outside formal educational settings. Having continuous access to support enabled them to set their own learning goals, focus on areas of weakness, and learn at a pace that suited their individual needs. As they became more confident in managing their learning, many reported taking greater initiative in exploring new language topics and monitoring their own progress.

Overall, the findings indicate that ChatGPT contributed to a stronger sense of learner autonomy. By giving students greater control over when, how, and what they learned, the tool appeared to support the development of self-directed learning habits and increased confidence in studying Arabic independently.

## 5 Discussion

This study set out to explore how non-native learners of Arabic perceive the use of ChatGPT as a tool for autonomous language learning. Overall, participants described ChatGPT as a helpful and accessible resource that supported different aspects of their learning, particularly vocabulary development, grammar understanding, reading comprehension, motivation, and independent learning. Although these findings broadly reflect trends reported in previous studies on AI-assisted language learning, the present study contributes a perspective that has received relatively limited attention: the experience of learners studying Arabic as a foreign language.

Vocabulary development emerged as one of the most noticeable benefits reported by participants. Many learners explained that interacting with ChatGPT exposed them to new words in meaningful contexts rather than in isolated lists. This appears to be particularly important in Arabic, where understanding a word often requires recognizing its relationship to a root and its various derived forms. The participants’ experiences therefore suggest that the value of ChatGPT may extend beyond introducing new vocabulary items; it may also help learners make sense of the relationships between words and their patterns of use. This interpretation is consistent with earlier research highlighting the importance of contextualized exposure for vocabulary learning (Boudouaia et al., 2024; Kohnke et al., 2023). At the same time, the findings indicate that learners are not merely memorizing vocabulary but actively engaging with language in ways that help them build connections between form and meaning.

The findings relating to grammar are equally noteworthy. Arabic grammar is often perceived by non-native learners as one of the most challenging components of language learning because of its complex inflectional system and reliance on grammatical case marking. Participants repeatedly emphasized that ChatGPT made difficult grammatical concepts more understandable by providing immediate explanations and examples. While this supports earlier arguments regarding the pedagogical value of AI-generated explanations (AlAwfi, 2024), it also raises an important question. Are learners actually developing a deeper understanding of grammatical structures, or are they simply finding the explanations easier to follow? The distinction is important because perceived understanding does not necessarily translate into long-term learning. The simplicity and accessibility of ChatGPT’s explanations may contribute to learners’ confidence, yet confidence alone cannot be taken as evidence of grammatical mastery. This is particularly relevant to autonomous learning because it reduces learners’ dependence on teacher intervention and allows them to address linguistic difficulties independently. The findings therefore suggest that the contribution of ChatGPT extends beyond grammar support itself to facilitating greater learner control over the learning process.” Future research will therefore need to examine whether these positive perceptions are reflected in measurable improvements in language performance.

A similar tension can be observed in the findings related to reading comprehension. Participants described ChatGPT as a useful support when working with Arabic texts, particularly when unfamiliar vocabulary or complex expressions interrupted their understanding. Rather than abandoning difficult texts, learners were able to ask questions, request clarification, and receive immediate assistance. This appears to reduce one of the major barriers faced by many language learners: the frustration that often accompanies reading authentic materials. Interestingly, some participants also reported using ChatGPT to explore cultural and literary meanings embedded within texts. This suggests that the tool may support a deeper level of engagement than simple comprehension. However, there is also a potential downside. If learners become accustomed to relying on AI-generated interpretations whenever they encounter difficulty, they may spend less time developing the analytical and inferential skills that effective reading normally requires. In this sense, ChatGPT can function either as a scaffold that promotes learning or as a shortcut that limits intellectual engagement, depending on how it is used.

Motivation emerged as another prominent theme throughout the study. Participants frequently referred to the convenience and availability of ChatGPT, noting that they could use it whenever they wanted without needing to wait for a teacher or language partner. This constant accessibility appears to have encouraged more frequent engagement with Arabic learning. Such a finding is especially significant because opportunities for authentic language practice are often limited for non-native learners of Arabic. Unlike classroom instruction, which is restricted by time and place, ChatGPT provides a learning environment that is continuously available. Nevertheless, increased participation should not automatically be interpreted as increased learning. Learners may spend more time interacting with the language simply because the experience is enjoyable and convenient. While this is undoubtedly beneficial, motivation alone cannot guarantee meaningful progress. The challenge for educators is therefore to harness the motivational advantages of AI tools while ensuring that learners remain focused on clearly defined learning goals.

The importance participants attached to immediate feedback further highlights the appeal of ChatGPT as a learning resource. Many learners valued the ability to receive corrections and explanations at the exact moment they encountered a problem. This immediacy creates a learning experience that differs substantially from traditional educational settings, where feedback is often delayed. At the same time, the quality of instant feedback should not be assumed to be equivalent to the quality of expert feedback. Unlike human teachers, AI systems may overlook contextual factors, fail to recognize subtle learner difficulties, or occasionally provide inaccurate responses. Consequently, the speed of feedback should be viewed as one of ChatGPT’s strengths, while its reliability remains something that learners need to evaluate critically.

The findings also have important implications for Arabic language pedagogy. Rather than viewing ChatGPT as a replacement for teachers, the results suggest that it may be most effective when used alongside traditional forms of instruction. Teachers can encourage learners to use ChatGPT for additional practice, revision, and exploration while also helping them evaluate the quality of the information they receive. In this way, AI becomes a tool that extends learning opportunities beyond the classroom rather than one that replaces established pedagogical practices. The increasing presence of AI technologies in education also highlights the importance of developing learners’ critical digital literacy. Students need to know not only how to use AI tools but also how to question, verify, and evaluate the information they generate.

Despite the positive views expressed by participants, the study also points to several challenges that should not be overlooked. Concerns about the accuracy of AI-generated information remain particularly relevant in the case of Arabic, where language technologies are generally less developed than in English. Errors in grammar explanations, translations, or cultural interpretations can potentially mislead learners if outputs are accepted without verification. Another concern involves over-reliance on AI support. The more learners depend on instant answers, the less likely they may be to engage in independent problem-solving or critical reflection. These issues suggest that successful integration of ChatGPT into language learning requires a balanced approach that combines the advantages of AI with appropriate pedagogical guidance and learner awareness.

In conclusion, the findings indicate that ChatGPT has considerable potential to support autonomous Arabic language learning. Its value appears to lie not only in the information it provides but also in its ability to create opportunities for continuous practice, exploration, and engagement. However, the benefits reported by participants should be interpreted in light of the study’s reliance on self-reported perceptions. Ultimately, the findings suggest that ChatGPT is most effective when treated as a learning companion rather than an authoritative source of knowledge. Used thoughtfully and critically, it can enrich the language-learning experience while complementing the guidance provided by teachers and formal instruction.

## 6 Conclusion

This study explored how non-native learners of Arabic experience the use of ChatGPT as a tool for autonomous learning. The participants generally viewed ChatGPT as a helpful resource that supported different aspects of their language learning, particularly vocabulary development, grammar learning, and reading comprehension. The ability to receive immediate explanations and feedback appeared to make learning more accessible and less dependent on teacher support, allowing learners to address difficulties whenever they arose. In this way, ChatGPT functioned not only as a source of information but also as a tool that supported greater learner autonomy and self-direction.

An important finding was the role ChatGPT played in encouraging learners to take greater responsibility for their own learning. Participants described using the tool not only to answer questions but also to explore language independently, practice at their own pace, and revisit areas they found challenging. For many learners, this flexibility seemed to increase both their engagement and their confidence. Rather than being limited to classroom activities, learning could continue whenever support was needed.

The study also sheds light on the growing role of AI in Arabic language education. Much of the existing research on AI-assisted language learning has focused on English and other widely taught languages. By examining learners of Arabic, this study provides evidence that similar benefits may be observed in a different linguistic context, despite the particular challenges that Arabic presents for non-native learners.

At the same time, the findings should not be taken to mean that ChatGPT can replace teachers or formal instruction. Participants valued the support provided by the tool, but effective language learning still depends on guidance, practice, and opportunities for meaningful interaction. ChatGPT appears to be most useful when viewed as a supplementary resource that works alongside established teaching practices rather than as an alternative to them.

Overall, the findings point to the potential of ChatGPT to support autonomous Arabic language learning. Its value lies not only in the information it provides but also in the opportunities it creates for learners to engage with the language more independently and consistently. As AI technologies become increasingly present in educational settings, understanding how they can be used in ways that genuinely support learning will become even more important.

## 7 Limitations and future research

The findings of this study should be considered in light of several limitations.

First, the study involved a relatively small number of participants. Although the sample provided rich insights into learners’ experiences, it does not allow for broad generalizations about all non-native learners of Arabic. Different learner populations may use ChatGPT in different ways or report different experiences.

Second, all participants were drawn from a single institutional context. Learning environments vary considerably in terms of teaching approaches, technological resources, and learner backgrounds. As a result, the experiences reported in this study may reflect characteristics of the specific context in which the research was conducted.

Another limitation concerns the type of data collected. The study relied mainly on participants’ own accounts of how ChatGPT influenced their learning. While these perceptions are valuable because they reveal how learners make sense of their experiences, they cannot confirm whether actual improvements in language proficiency occurred. What learners believe has helped them may not always correspond to measurable learning gains.

The study was also limited by the absence of additional data sources. Classroom observations, language assessments, learner journals, or analysis of students’ language performance could have provided a more complete understanding of ChatGPT’s impact. The use of multiple sources of evidence would have strengthened the study by allowing learners’ perceptions to be compared with observable learning outcomes.

Building on these findings, future research could examine larger and more diverse groups of Arabic learners to determine whether similar patterns emerge across different contexts. Studies conducted across multiple institutions would also help identify how local educational conditions influence the use of AI tools for language learning.

There is also considerable scope for mixed-methods research that combines learners’ experiences with objective measures of language development. Such studies could provide a clearer picture of the relationship between perceived benefits and actual learning outcomes. In addition, future research could compare learners who use ChatGPT with those who rely on more traditional learning resources in order to better understand the specific contribution of AI-assisted learning.

Finally, longitudinal research is needed to explore the long-term effects of ChatGPT use. The present study captures learners’ experiences at a particular point in time, but language learning is a gradual process that unfolds over months or years. Future studies could investigate whether the motivation, autonomy, and perceived learning benefits reported here are sustained over time and whether they lead to measurable improvements in Arabic language proficiency.

## Data Availability

The original contributions presented in the study are included in the article/supplementary material, further inquiries can be directed to the corresponding author.
